# A Comparative Evaluation of Regulatory T Cells Profile among Acute and Chronic Cutaneous Leishmaniasis Using Flow Cytometry

**Published:** 2019

**Authors:** Fahimeh FIROUZJAIE-KARDER, Behnaz AKHOUNDI, Mehdi MOHEBALI, Farid AZMOUDEH ARDALAN, Abbas RAHIMI-FOROUSHANI, Fatemeh MESGARIAN, Homa HAJJARAN, Hossein MORTAZAVI, Yadollah SHAKIBA, Sorour CHAREHDAR, Samira ELIKAEE, Zahra KAKOOEI, Zahra SHAFEGHAT

**Affiliations:** 1. Department of Medical Parasitology and Mycology, School of Public Health, Tehran University of Medical Sciences, Tehran, Iran; 2. Center for Research of Endemic Parasites of Iran (CREPI), Tehran University of Medical Sciences, Tehran, Iran; 3. Department of Pathology, Imam Khomeini Hospital, Tehran University of Medical Sciences, Tehran, Iran; 4. Department of Biostatistics and Epidemiology, School of Public Health, Tehran University of Medical Sciences, Tehran, Iran; 5. Gonbade Kavous Health Center Laboratory, Golestan University of Medical Sciences, Gonbade Kavous, Iran; 6. Department of Dermatology, Razi Hospital, Tehran University of Medical Sciences, Tehran, Iran; 7. Regenerative Medicine Research Center, Kermanshah University of Medical Sciences, Kermanshah, Iran

**Keywords:** Cutaneous leishmaniasis, Regulatory T cells, Flow cytometry

## Abstract

**Background:**

Cutaneous leishmaniasis (CL) is described as a major health problem in many countries of the world. Regulatory T cells (Tregs) are characterized as one of immunologic indexes. One of the best methods to determine of Tregs percentage is flow cytometry. The aim of this study was determination of the role of Tregs profile among acute and chronic forms of human CL using flow cytometry analysis.

**Methods:**

This study was conducted on 24 patients referred to Laboratory of Leishmaniasis, Tehran University of Medical Sciences, Tehran, Iran with acute and 14 patients with chronic phases of CL as well as 15 healthy individuals as control group in 2015–2016. After microscopic examination, 2 ml of peripheral blood samples were collected for determining percentage of CD_4_^+^ CD_25_^+^ CD_127_ low Tregs by using flow cytometry method.

**Results:**

Using flow cytometry analysis, the average percentage of Tregs were calculated 5.73, 6.71 and 6.61 for acute, chronic and healthy individuals, respectively. With SPSS software and Scheffe multiple comparison tests, the differences within in these groups are statistically significant (*P*=0.04) and between the acute and chronic group, there was marginally significant with approximately 91% of confidence level (*P*=0.088).

**Conclusion:**

Marginally differences were found significantly among averages of Regulatory T cells, acute and chronic phases of CL. Further comprehensive studies can be needed to verify the role of Tregs in both phases of CL cases.

## Introduction

*L*eishmaniasis, known as a sever health problem in many countries of the world, is caused by the genus *Leishmania*. A wide spectrum of clinical diseases such as self-healing skin lesions, cutaneous, mucocutaneous and visceral form with high rate of mortality results from *Leishmania* parasite (1). The most reports (more than 90%) of cutaneous leishmaniasis are from Iran, Syria, Afghanistan, Saudi Arabia, Brazil and Peru (2). The causative agents of CL in Iran are *L. major* and *L. tropica*, of course, the first one is the most common in endemic area (3).

The lesions from *L. major* often ameliorate in 3–4 months and they have more inflammation whereas *L. tropica* causes lesions, which have longer duration and less ulceration (4). Severity of lesions depends on multiple parameters such as species of parasite, the host immunity, and the environment. Several immune mechanisms involve in control of the infection of *Leishmania* parasite and it seems a combination of TH_1_, TH_2_ immune response is deal with leishmaniasis (5–8). CD_4_ T cells are the main sources of INF-γ and secretion of INF-γ can activate macrophages and result in killing the amastigotes (9). However, a small number of parasites may remain and result in reinfection (10–12).

Natural Regulatory T cells (n Tregs) as the main subset of Tregs in immune system, are characterized by CD4^+^, CD25^+^, CD127 low Foxp3^+^ and play an important role in regulation of immune system and prevent from excessive pathological damage during inflammatory responses (13–15). On the other hand, Tregs contribute to parasite persistence through the prevention of its clearance (16). Therefore, the exact role of these cells during *Leishmania* infection has not been characterized. To achieve this purpose some efforts have been done on animal model (17–20) and also some studies have focused on the role of Tregs at the site of lesions (21) so few information are available in determination of Tregs role in *Leishmania* infection.

We aimed to investigate and quantify the Tregs in the peripheral blood of patients who were suffering from acute and chronic leishmaniasis compared to healthy people who never resident in endemic area by using specific surface markers like CD_4_, CD_25_ and CD_127_.

## Materials and Methods

### Patients with CL and healthy control subjects

This study was conducted on 24 patients referred to Laboratory of Leishmaniasis, Tehran University of Medical Sciences, Tehran, Iran in 2015–2016.

Three groups of people were selected. In the acute group consisted 24 patients (17 males, 7 females, age range: 4–75 yr old) with active CL lesions whom their lesions’ duration were less than 6 months and they response to systemic or intralesional administration of meglumin antimoniate (Glucantime^®^). The chronic group included 14 patients (9 males, 5 females, age range: 6–74 yr old) with unresponsive to complete systemic treatment with at least two full courses of intramuscular administration of Glucantime^®^ and their lesion’s did not heal after 6 months.

The control group included 15 healthy people (9 males, 6 females, age range: 7–71 yr old) from Tehran city (capital of Iran as non-endemic area) without any ulcer of *Leishmania* infection in their body, and any trip to endemic areas in 2 months ago. On the other hand, they were without any specific disease such as HIV^+/^AIDS, renal failure, cancer and pregnancy and did not use immune suppressive drugs.

### Parasitology exam (golden standard method)

Firstly, the surface of the patient’s lesions was disinfected by ethanol 70°; then some smears were prepared from the secretion of around the lesions with vaccinostyle equipment. Serosity materials of several parts of each lesion were prepared on thin slides, and after fixation by methanol and staining by Giemsa the amastigotes were investigated by microscope.

### Isolation of Tregs and Flow cytometry

After confirmation of amastigotes by parasitological exam in the leishmaniases laboratory, School of Public Health, Tehran University of Medical Sciences; the peripheral blood samples (2ml) were collected in tubes containing EDTA. Anti-human CD_4_-FITC/CD_25_-PE (eBio science), Anti human CD_127_-PE cyanin S (e Bioscience) and whole blood sample were utilized for flow cytometry analysis in the Medical Center of Pediatric of Tehran, Tehran University of Medical Sciences. According to antibodies protocols, blood added to the tube including antibodies and incubated for 30 min at 4°C. Then lyses procedure was done and finally cell acquisition was performed on a BD Accuri C6 flow cytometer.

### Ethical approval

Written informed consent was obtained from the adult patients or the parents of the children. This study was approved by the Research Ethical Review Committee of Tehran University of Medical Sciences, Tehran, Iran (Code no: 93-01-27-25290).

### Statistical analysis

Finally, due to confirmation, the samples were sent to reliable related center for sequencing. The SPSS software (ver. 22, Chicago, IL, USA) was used for statistical analysis and Tregs normality distribution were assessed by Kolmogorov-Smirnov test. For normal data distributions, the student’s 1-test and ANOVA followed by Scheffe multiple comparison tests were used and *P*-values of less than 0.05, and between 0.05 and 0.1 were considered significant and marginally significant, respectively.

## Results

Thirty-eight leishmaniasis patients (24 acute, 14 chronic) and 15 healthy persons participated in this investigation.

In terms of number of lesions in patients groups, 25 patients (65.8%) had one lesion and 13 patients (34.2%) had two or more than two lesions. Moreover, these lesions were on face, hand, foot and other organs in 10 (26.3%), 14 (36.8%), 6 (15.8%), and 8 (21.1%) patients, respectively.

**Fig. 1:**
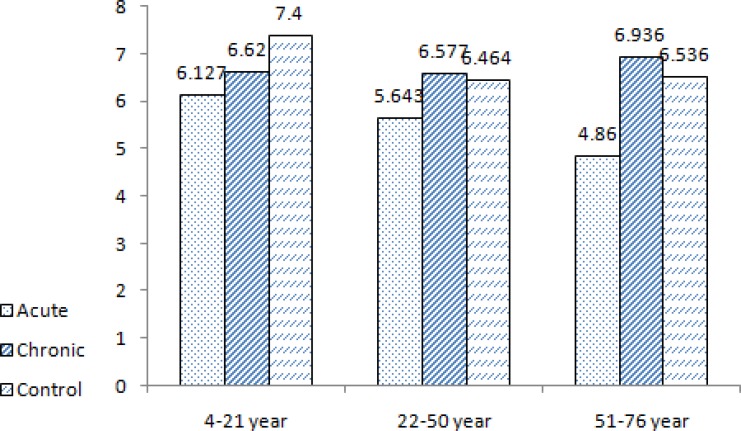
The comparison between percentages of Tregs in acute and chronic CL and control with age groups

Based on the Tregs percentage’s mean; there were no significant differences in age groups, gender, location, number, and duration of lesions in disease groups. For instance, the percentages of Tregs in order to age groups were shown in Fig. 1 and *P*=0.809 proves that the difference in age was not significant.

High expression of CD_25_, expression of Foxp3, GITR, CTLA4. CD_103_ and low expression of CD_127_ are characterized as Tregs markers. Therefore, in this study, the frequency of Tregs was determined via the evaluation of CD_4_^+^ CD_25_^+^ CD_127_^low^ cells percentage in peripheral blood by flow cytometry.

The Tregs percentages’ mean for acute, chronic and healthy groups was 5.73, 6.71 and 6.61 respectively.

After statistical analysis, the difference of Tregs’ mean was significant within the group (*P*=0.04). Then the comparison performed between the groups so there were no significant differences between acute and control (*P*=0.126) and between chronic and control groups (*P*=0.979). However, the difference between acute and chronic groups was marginally significant with approximately 91% of confidence level (*P*=0.088). Moreover, the comparison of acute, chronic CL and control (healthy) group’s numbers between Tregs percentages’ mean and themselves was shown in Table 1.

**Table 1:** The comparison of acute, chronic CL and control (healthy) group’s numbers between Tregs percentages’ mean and themselves

***Group type***	***Mean Difference***	***Std Error***	***P-value***
Acute Chronic	−0.985	0.43	0.088
Acute Control	−0.886	0.42	0.126
Chronic Control	0.098	0.48	0.979

## Discussion

Regulatory T cells play an important role in regulation of immune response and is responsible for immunologic tolerance. Tregs can prevent from excessive pathological damage and control the infectious agents by modulating of effector T cells (13–15). These cues can help to parasite persistence in mouse model with *L. major* and down regulates both TH_1_ and TH_2_ immune responses (16). Tregs are characterized by high expression of CD_25_, expression of Foxp3, GITR, CTLA_4_, CD_13_ and low expression of CD_127_, one of the best techniques for evaluation of these cells is flow cytometry.

Over the years, numerous studies have been carried out on the Tregs evaluation of laboratory animals (17, 22–24). Some of the human studies have been assessed with the other techniques or at lesion site or based on Leishmanin skin test (LST), so there are a few investigation on peripheral blood and because the relation between Tregs and clinical forms of cutaneous leishmaniasis is not so clear, therefore in order to better identification of Tregs’ role, we classified the people into three groups included acute, chronic and control groups (27, 28).

We assessed the frequency of CD_4_^+^ CD_25_^+^ CD_127_^low^Tregs in peripheral blood of patients and control group. The difference of Tregs percentage between acute and chronic group is marginally significant (*P*=0.088).

In India, spleen aspiration and peripheral blood of patients with visceral leishmaniasis and healthy people who were living in endemic area were investigated and observed no increasing in Foxp3 in patients. Our specimen was only peripheral blood and against the research of Maurya, we found marginally significant difference between acute and chronic patients (25). In the other study in India, two groups included the patients infected by *L. donovani* and control group were selected and surveyed their peripheral blood and bone marrow and found a large amount of Tregs in patient’s bone marrow and realized that the patients with increase in IL-10 do not response to treatment (26). In present study, cutaneous leishmaniasis (*L. major*) was investigated so the amount of Tregs in acute, chronic and control group were determined. On the other hand, Foxp3 gene expression and also IL 10, IFN-*γ* and IL4 were investigated and increasing of these factors was significant in patients group. However, in this study, determination of Tregs has been performed by using CD4, CD25 and CD127 and marginally significant difference was obtained between acute and chronic group.

In Iran, the regulatory T cells profile in early and late lesions of cutaneous leishmaniasis resulted from *L. major* and after collecting the skin biopsy, RT-PCR and immune fluorescent staining were performed they observed increase in mRNA Foxp3 expression and protein staining of Tregs, markers in chronic biopsy samples (27). Our study was performed only on peripheral blood not skin biopsies and acute and chronic groups were compared with controls. However, there were not any control group. Furthermore, they used immunofluorescence assay but the method used in our study for evaluation of Tregs was flow cytometry.

In another study in Colombia, two groups were described: first the people without any ulcer but their Leishmanin skin test were positive, the other one, infected by *L. panamensis* the amount of CD_4_^+^ CD_25_^+^ CD_127_^low^Tregs were determined in peripheral blood and IL-10, INF-γ and Foxp3 expression were evaluated in skin biopsies and the result indicated that the second group had more Tregs in their blood and lesions (28). In this study, the patients’ classification was based on the duration of ulcer not LST’s results and the Tregs percent were compared between the acute, chronic and control groups. While Rodriguez-Pinto et al. followed up the patients during treatment and evaluated Tregs with both flow cytometry and Foxp3 gene expression. We performed only flow cytometry method but the results of both studies were similar and obtained significant and marginally significant difference between chronic and healthy people in their study and acute & chronic in our study, respectively.

In Brazil, the people were classified in to three groups based on infecting by *L. braziliensis* including cutaneous leishmaniasis infected patients (CL), sub cutaneous infected patients (SC) and uninfected control (UC). The amount of Tregs were similar in every three groups but IL-10 production and lymphocyte proliferation in CL was more than the other groups, so they said that increase in Tregs function may be result in impairment in elimination of the parasite (29).In present study, we had increase in amount of Tregs in chronic group and comparison of Tregs between acute and control was not significant.

In Iran, Tregs percent in three resident groups in endemic area were investigated: 1-the recovered patients with positive LST, 2-uninfected persons with positive LST and 3-uninfected persons with negative LST. They evaluated Tregs in peripheral blood and also IL-10, TGF-β- INF-γ by ELISA Technique and Foxp3 expression by RT-PCR, the frequency of Tregs were similar in 3 groups more over Foxp3 expression in second group was more than the other group and in first group, the amount of IL-10 and INF-γ were the most (30). Whereas in our study, the amount of Tregs in chronic group was more than acute group.In addition, our findings showed that the age group, sex, the number of lesions and their location in patient’s body had no role in amount of Tregs percent.

There were some limitations in this study such as requirement to advanced flow cytometry instrument because of low amount of regulatory T cells percentages in whole blood and lack of access to chronic patients.

In this study, the inhibitory roll of Tregs in immune response was proceed and flow cytometry was mentioned as a practical technique in different researches such as parasitology and during this, the mean percentage of Tregs were estimated 5.73, 6.71 and 6.61 for acute, chronic and healthy group respectively. After entering the data in SPSS software and statistical analysis followed by Scheffe multiple comparison tests, the differences between the acute and chronic group, was marginally significant (*P*=0.088).

## Conclusion

Marginally differences were found significantly among averages of Regulatory T cells, acute and chronic phases of CL. Further comprehensive studies can be needed to verify the role of Tregs in both phases of CL cases.
